# Contrasting neurofunctional correlates of face- and visuospatial-processing in children and adolescents with Williams syndrome: convergent results from four fMRI paradigms

**DOI:** 10.1038/s41598-024-60460-5

**Published:** 2024-05-05

**Authors:** Madeline H. Garvey, Tiffany Nash, J. Shane Kippenhan, Philip Kohn, Carolyn B. Mervis, Daniel P. Eisenberg, Jean Ye, Michael D. Gregory, Karen F. Berman

**Affiliations:** 1 Section on Integrative Neuroimaging, Clinical and Translational Neuroscience Branch, National Institute of Mental Health, Intramural Research Program, National Institutes of Health, 10 Center Drive, Bethesda, MD 20892 USA; 2 Department of Psychiatry, University of Cambridge, Cambridge, UK; 3 Georgetown University School of Medicine, Washington, DC 20007 USA; 4 Neurodevelopmental Sciences Laboratory, Department of Psychological and Brain Sciences, University of Louisville, Louisville, KY 40292 USA

**Keywords:** Neurodevelopmental disorders, Perception, Object vision, Chromosome abnormality

## Abstract

Understanding neurogenetic mechanisms underlying neuropsychiatric disorders such as schizophrenia and autism is complicated by their inherent clinical and genetic heterogeneity. Williams syndrome (WS), a rare neurodevelopmental condition in which both the genetic alteration (hemideletion of ~ twenty-six 7q11.23 genes) and the cognitive/behavioral profile are well-defined, offers an invaluable opportunity to delineate gene-brain-behavior relationships. People with WS are characterized by increased social drive, including particular interest in faces, together with hallmark difficulty in visuospatial processing. Prior work, primarily in adults with WS, has searched for neural correlates of these characteristics, with reports of altered fusiform gyrus function while viewing socioemotional stimuli such as faces, along with hypoactivation of the intraparietal sulcus during visuospatial processing. Here, we investigated neural function in children and adolescents with WS by using four separate fMRI paradigms, two that probe each of these two cognitive/behavioral domains. During the two visuospatial tasks, but not during the two face processing tasks, we found bilateral intraparietal sulcus hypoactivation in WS. In contrast, during both face processing tasks, but not during the visuospatial tasks, we found fusiform hyperactivation. These data not only demonstrate that previous findings in adults with WS are also present in childhood and adolescence, but also provide a clear example that genetic mechanisms can bias neural circuit function, thereby affecting behavioral traits.

## Introduction

Visual processing requires integration of a rich variety of stimulus types and features, including color, form, and orientation in space. Two key domains within higher-order visual processing that not only may have had different evolutionary repercussions for hominid speciation^1^, but also are argued to have different developmental trajectories in modern humans (vide infra, “Discussion” section)^2,3^, are the processing of socially salient stimuli and the processing of spatial information. The former, particularly the ability to detect and recognize human faces, is essential for successfully managing our social world, whereas visuospatial judgments are necessary for navigating our physical environment. Altered abilities in both of these visual domains have been reported in neuropsychiatric disorders such as autism and schizophrenia^4–7^ and may be associated with symptom severity^8–10^ and treatment outcomes^11^, but elucidating underlying mechanisms is complicated by the inherent clinical and genetic heterogeneity of these conditions. While the specific molecular and neurobiological etiologies of these complex visual processing phenotypes may be distinct across disorders, establishing a mechanistic foothold on clinically apparent alterations in human visual processing may open new routes to discovery that are widely applicable. The study of a syndrome with a well-defined genotype and a characteristic clinical/cognitive phenotype, such as Williams syndrome, offers an incisive opportunity in this regard.

Williams syndrome (WS) is a rare, genetic, neurodevelopmental condition with estimated prevalence of 1 in 7500 live births^12,13^, resulting from a well-documented hemizygous deletion of ~ 1.5 megabases of DNA at chromosomal locus 7q11.23 harboring ~ 26 genes^13^. A physical map of the WS copy number variant (CNV) was reported in 1998 using fluorescence in situ hybridization^14^ and has been the focus of careful study with molecular genetic methods since. Because this 7q11.23 portion of the genome, referred to as the Williams syndrome critical region, is flanked by low copy repeat segments of DNA, it is prone to de novo (or Mendelian inherited) errors during homologous recombination and is, thus, unusually stereotyped among human genetic conditions. The fact that the WS 7q11.23 CNV has similar breakpoints in more than 95% of people with WS^15^ facilitates the study of links between genotypes and phenotypes.

Clinically, WS is typified by specific and sensitive personality and cognitive profiles^16,17^, the hallmarks of which are, respectively, increased social drive (often termed “hypersociability” or “social disinhibition”) and severe visuospatial construction deficits. Early descriptions of the peaks and valleys of neurocognitive capabilities in WS, including seminal work by Bellugi et al.^18^, contrasted impaired function on visuospatial tests, such as recognizing line orientation, with relatively spared face processing, particularly for face recognition. The latter has been an area of specific interest, in light of the sociability of people with WS and their remarkably increased interest in, and gaze preference for, faces^17,19–22^, even in infancy^20^. Considerable further research has clearly and consistently corroborated the presence of visuospatial construction deficits as a core cognitive feature of WS^13,18,23–27^. However, reports of face processing capacities have been more nuanced, with some documenting largely intact abilities in people with WS^18,28–32^, while others have identified altered performance^26,33–35^, often varying as a function of specific task demands and stimulus types.

Efforts to relate these contrasting cognitive features to neural structure and function in WS have paralleled the behavioral findings: Alterations in areas classically linked to spatial processing have been consistently reported, whereas the neuroimaging literature regarding circuits classically related to face processing has been mixed^36^. Regarding the former, there has been substantial agreement across a number of studies that all show altered structure and function of the intraparietal sulcus (IPS), which is located within the inferior parietal lobule^37^ and is a key hub for visuospatial processing abilities that include navigating through space and understanding the spatial properties of objects^38^. Specifically, decreased IPS gray matter volume and macrostructural alterations^39–43^, including reduced IPS folding and sulcal depth, in WS have been well documented^44,45^. Neurofunctionally, task-based fMRI during visuospatial construction has revealed hypoactivation in the IPS, and differences in IPS functional connectivity have also been identified^46,47^. Whether these adult IPS functional phenotypes also are consistently evident in childhood or adolescence is unknown. Nonetheless, the IPS appears both structurally and functionally affected in people with WS and likely plays a central role in the visuospatial behavioral problems associated with the syndrome.

With respect to face processing, akin to the behavioral literature, neuroimaging experiments in WS have yielded more nuanced results. While some structural MRI studies in WS have reported increased gray matter volume, density, and thickness in the fusiform gyrus^48–50^, a key region subserving social and face processing^51^, others have found preserved or even reduced fusiform gray matter volume^52–54^. Similarly with functional imaging, one previous fMRI study identified increased activation of fusiform regions in WS during face matching^55^, whereas three others found that fusiform activation in adults with WS remained relatively intact^39,56,57^. Thus, further work is needed to better understand fusiform function in WS, and characterization in younger cohorts is especially needed.

The contrasting findings—both behavioral and neuroimaging—regarding visuospatial versus face processing in individuals with WS lend themselves to consideration through the lens of the dorsal and ventral cortical visual processing stream framework first introduced by Ungerleider and Mishkin^58^. As shown in this seminal work, the cortical networks that subserve visual processing split into two pathways after emerging from primary visual cortex: the “where” pathway of the dorsal stream and the “what” pathway of the ventral stream^58^. The ventral “what” stream, which processes characteristics of objects, including faces^58–60^, traverses the ventral surface of the brain anteriorly from the occipital lobe through the inferior temporal lobe, including the fusiform gyrus. The dorsal “where” stream, which supports visuospatial processing^58,59^, traverses dorsally and anteriorly from the occipital lobe through the parietal lobe, including the IPS. In later refinements it has been suggested that the dorsal stream also supports neural representations of “how” an object is used and is integral to visually-guided behavior^61–63^. In considering WS within this two-stream visual processing framework, the contrasting visuospatial performance deficits and behavioral findings during face processing in WS would point to differential neural alterations within the dorsal versus ventral streams. And indeed, as described above, prior literature consistently suggests under-activation of dorsal stream regions in WS, whereas there is less consensus about alterations in face processing abilities and ventral stream activity.

Given the conceptual intersection between the behavioral contrast of face recognition and visuospatial processing in WS on the one hand, and the ventral and dorsal stream neural circuits that classically underly these cognitive domains on the other hand, in the present study we explored visuospatial processing and face processing in children and adolescents with WS through the use of two independent fMRI tasks for each and tested for convergence across tasks probing similar cognitive domains. We hypothesized that, compared to age- and sex-matched typically developing children and adolescents (TDs), those with WS would show hypoactivation of regions typically engaged during visuospatial processing tasks, and, in contrast, we expected hyperactivation of regions typically recruited for face processing. Additionally, because considerably less neuroimaging work has been carried out in children and adolescents with WS than in adults, we sought to extend previous adult findings to this critical developmental period.

## Methods

### Participants

As part of the National Institute of Mental Health Intramural Research Program Study of Brain Development in Williams Syndrome, children and adolescents with WS (age range 7–19 years) traveled with their families to the NIH Clinical Center in Bethesda, MD, to participate in a protocol involving detailed documentation of history, physical examination, radiological screening for brain structural and cerebrovascular abnormalities, investigational structural and functional MRI scanning, neuropsychological testing, and genetic research. All participants were physically healthy and had no clinical abnormalities on structural MRI and no clinically significant alterations in cerebral vasculature based on MR angiography, both read by staff neuroradiologists.

To maximize compliance with study procedures and minimize the possibility that results were unduly biased by group differences in medical or cognitive factors, there were several special inclusion criteria for participants with WS. First, we recruited particularly rare participants with WS who are not only physically healthy—having no current cardiovascular or other vascular problems that are common to this copy number variant group due to hemideletion of the *Elastin* gene—but who also have no other medical problems that could affect interpretation of the neuroimaging results. MR angiography, a procedure that is unusual in the literature and in the community, was performed on site, both as a service to our participants by ensuring the integrity of the cerebral vasculature and to ensure the integrity of our neurofunctional research measures. Second, we recruited participants with WS who are from the higher end of the cognitive ability range for the syndrome (normal to low-normal average IQ).

Typically developing, physically healthy children and adolescents were also recruited as a comparison group and were included in the protocol if they had no history of psychiatric, neurological, or medical disorders, were free from conditions that would make MRI scanning unsafe, and were able to tolerate MRI scanning (i.e., no claustrophobia). For between-group analyses each participant with WS was age- and sex- matched to two TD participants. All participants provided written and verbal assent, and their parents/guardians provided written and verbal consent. All study procedures were approved by the NIH Institutional Review Board. Demographic information for participants in each of the four separate task-based fMRI sequences is shown in Table 1.

**Table 1:** Participant demographics.

Task category	Task name	Participant group	n	Mean age (years)	Age range	Sex (M/F)
Face Processing	Match-to-Sample Face Task	Williams syndrome	20	12.45 ± 3.35	7.5–19.0	5/15
		Typically developing	40	12.53 ± 3.63	6.6–18.4	10/30
	One-Back Face Matching Task	Williams syndrome	21	12.50 ± 3.21	7.5–19.0	5/16
		Typically developing	42	12.32 ± 3.53	6.6–18.4	10/32
Visuospatial Construction	Tetris Task	Williams syndrome	16	12.97 ± 2.78	7.5–17.6	3/13
		Typically developing	32	12.87 ± 3.33	7.5–18.4	6/26
	Spatial Location Task	Williams syndrome	18	13.81 ± 3.05	9.7–18.2	4/14
		Typically developing	36	12.89 ± 3.43	7.5–18.0	8/28

Age and sex of participants included in the four fMRI task analyses, grouped into the broader categories of face processing (Match-to-Sample Face Task and One-Back Face Matching Task) and visuospatial processing (Tetris Task and Spatial Location Task). Each participant with Williams syndrome was age- and sex- matched to two typically developing participants.

### Neuropsychological testing

Participants with WS and TDs were administered the Kaufman Brief Intelligence Test, Second Edition (K-BIT2)^64^ to determine a composite Intelligence Quotient (IQ) score for each person.

### Scan acquisition and functional tasks

**Figure 1:**
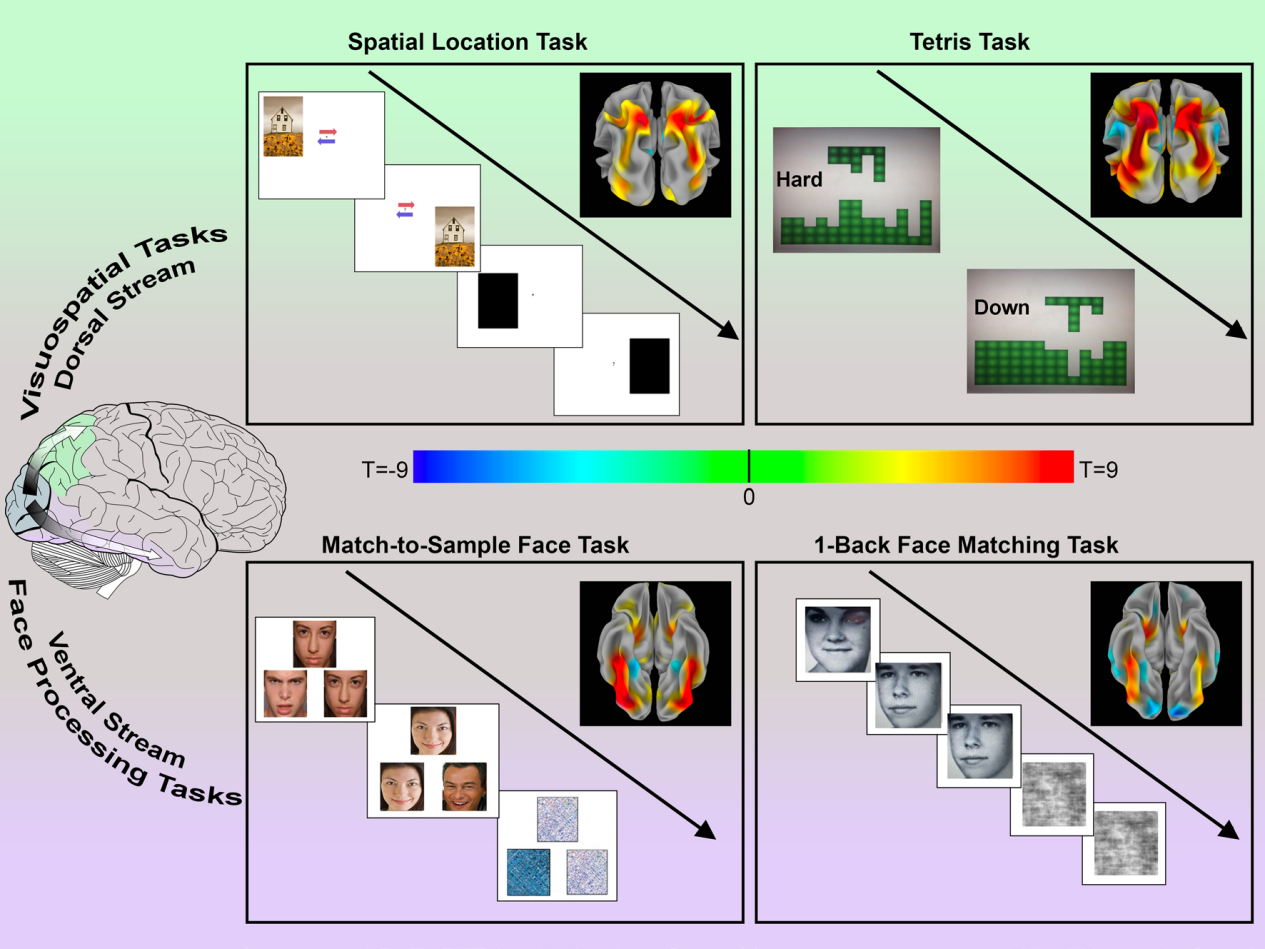
fMRI paradigms used to investigate visuospatial and face processing and activation patterns in typically developing participants. Top and bottom sections show fMRI tasks used to study, respectively, visuospatial processing within the dorsal stream and face processing within the ventral stream, along with activation patterns for each task in our cohort of typically developing children thresholded at *p* < 0.001, FDR corrected; data are shown on inflated brain renderings with posterior views for the dorsal stream and inferior views for the ventral stream. *Top left box* depicts the Spatial Location Task, in which individuals judged the height of consecutively presented stimuli or responded to a sensorimotor control condition consisting of black boxes that were always at the same height. *Top right box* depicts the stimuli for the Tetris Task in which individuals moved a puzzle piece to fit into the lower image; “hard” trials included a complicated “landscape”, while “down” trials only required simple straight-down movements. *Bottom left box* depicts the Match-to-Sample Face Task, in which three faces or scrambled images were concurrently presented and participants indicated which face on the bottom matched the image at the top. *Bottom right box* depicts the One-Back Face-Matching Task in which a series of faces or scrambled images was presented and participants indicated whether each image matched the image shown immediately prior. Note that for the two visuospatial tasks the activation patterns demonstrate robust activation of the dorsal stream, particularly the bilateral intraparietal sulci, and for the two face processing tasks the activation patterns demonstrate robust activation of the ventral stream, including the bilateral fusiform gyri. The image on the left depicting dorsal and ventral streams was adapted from: https://commons.wikimedia.org/wiki/File:Ventral-dorsal_streams.svg.

All participants underwent T1-weighted structural scanning and functional fMRI on the same 3T MR750 GE scanner using a 32-channel head coil. These procedures included T2*-weighted fMRI EPI sequences during task performance (1.88 × 1.88 × 3 mm voxel resolution, TR/TE 2000/24 ms, flip angle 77°) and a multi-echo MPRAGE sequence (resolution 1 × 1 × 1 mm, TR/TE 10.5/1.8 ms, flip angle 7°) for coregistration and to aid warping to standard anatomical space. During functional scanning, participants performed four separate tasks: two targeted at face processing and two targeted at visuospatial processing. For analysis of each fMRI paradigm, a condition of interest was compared to a matched sensorimotor control. For all tasks, participants responded by pushing one of four buttons: up, down, left, or right. Examples of face-processing and visuospatial stimuli presented during each task paradigm are shown in Fig. 1.

### Face processing tasks

The *Match-to-Sample Face Task* was based on a paradigm developed by Hariri et al.^65^ and has been previously described by Cole et al.^66^ At each trial, participants were shown three concurrent images: a target at the top of the screen and two images side-by-side below. For the active face matching task condition, images were of human faces. For the sensorimotor control condition, stimuli were scrambled face images. For both trial types, participants were instructed to identify which of the two lower images matched the target image at the top and then press a button to indicate their choice. Participants completed four runs of this task, with each 4-min run consisting of 2–5 alternating blocks of either faces or scrambled sensorimotor controls. Each block consisted of 1–4 trials preceded by a fixation cross presented for 2 s. Contrasts were created from a subtraction of beta maps based on stimulus-specific blocks: here, the active task condition (face stimuli) minus the control condition (scrambled images).

The *One-Back Face Matching Task* was based on an object processing localizer fMRI task from Chao and Martin^67^. Participants were sequentially shown pictures of tools and faces in black-and-white and were instructed to press a button if the currently-presented picture was the same as the previous picture in a one-back study design. Neutral conditions showed black-and-white scrambled images, and participants were again instructed to press the button each time they saw the same scrambled image twice in a row. Participants completed two runs (each 3 min 20 s long); each run consisted of three blocks of human faces, mechanical tools, scrambled faces, and scrambled tools. The number of trials in each block ranged from 4 to 12. Contrasts were created from a subtraction of beta maps based on stimulus-specific blocks: here, the active task condition (face stimuli) minus control condition (scrambled images).

### Visuospatial processing tasks

The *Tetris Task* was created in-house using Presentation software (Neurobehavioral Systems, Inc., Berkely, CA). Participants completed four runs of this task. Each run lasted 5 min and 30 s and consisted of three blocks each of 4–12 trials of “easy,” “hard,” and “straight down” conditions. For these tasks, participants were instructed to use a button box to move a shape constructed of squares to the left, right, and down to fit into a bottom space. Conditions were either easy, with only one spot to fit the puzzle piece, “straight down” for which the participant simply had to press the down button to fit the piece into one opening, or hard, where there were two similar appearing spots to fit the puzzle piece. Contrasts were created from a subtraction of beta maps based on stimulus-specific blocks: here, the active task condition (hard condition) minus the control condition (straight down).

For the active condition of the *Spatial Location Task*, created using EPrime software (Psychology Software Tools, Inc., Pittsburgh, PA), an image was presented on the left of the screen followed by an image on the right. Horizontal arrows shown mid-screen instructed participants to press the right button if the image on the right was at the same vertical height as the previously presented left image, or to press the left button if the right image was not at the same height as the first, left image. A sensorimotor control condition consisted of black boxes that were always at the same height and participants were instructed to always press the right button during these blocks. Participants completed two 5-min runs of this task, with each run consisting of six blocks. Contrasts were created from a subtraction of beta maps based on stimulus-specific blocks: here, the active task condition (location stimuli requiring vertical height determinations) minus the sensorimotor control condition (scrambled images always at the same height).

### Pre-scan participant training

Prior to fMRI during task performance, participants were trained outside of the scanner, first, with orientation to the response button box. Once participants mastered button presses, they underwent specific training for each task. They were taught about the Match-to-Sample Face Task, One-Back Face Matching Task, and Spatial Location Task via a Microsoft PowerPoint presentation, and for the Tetris Task via verbal instructions prior to and during practice of the task. Each participants then completed a practice round for each task while an experimenter observed the participant’s button presses to ensure full understanding. Training and practice were repeated as needed until the participant’s performance was above chance. Then, immediately prior to completing each task in the MRI scanner, participants were reminded of the instructions. During fMRI, an experimenter monitored task performance by observing participant responses on a screen and on a button box monitor in real time.

### fMRI processing

For all functional scans, the first five volumes were discarded, and subsequently each scan was slice-time corrected using AFNI’s^68^ 3dTshift and motion-corrected using SPM5^69^. Three T1-weighted MEMPRAGE sequences were collected, N3-intensity-normalized^70^, and averaged together using AFNI software^68^ to create a single average structural scan for each participant. All fMRI data were coregistered to each individual’s T1 structural image. A template T1-weighted structural scan was constructed using ANTs software^71^ to equally represent WS and TD children and to reduce confounding effects of spatial warping that may impact between-group analyses; the template was aligned to MNI space. Each individual’s T1 structural scan was then nonlinearly warped to the study-specific template and the warping parameters were carried through to the coregistered functional scan, followed by spatial smoothing using an 8 mm FWHM kernel. fMRI volumes corrupted by motion-related or other artifacts were excluded from further analyses using ART software^72^ if global signal of the timepoint was greater than nine standard deviations from the mean across time or if motion between volumes exceeded 1.5 mm. Participant data were excluded if motion was above 0.4 mm mean framewise displacement after ART scrubbing. SPM5 was used to generate first-level contrast maps for each task for each individual by creating a subtraction of beta weights while creating task regressors and accounting for hemodynamic response.

Prior to processing and quality control of the fMRI data, there were 22 total participants with WS who completed scanning. After preprocessing, 16 of those participants satisfactorily completed all four tasks and had useable fMRI data. Excessive motion during scanning and/or participant fatigue precluded inclusion of the data for all tasks for the remaining six participants. Of these six, one satisfactorily completed three of the four tasks, three completed two, and two individuals each had one usable task. After quality-controlled data were identified, two TD controls were age- and sex-matched for each participant with WS for each task.

### Statistical analyses

First, general linear modeling, controlling for chronological age and sex, was carried out using AFNI’s 3dttest++ within the group of TD participants for each of the four task paradigms separately to confirm that each paradigm activated expected brain regions. Next, to test for significant differences between the WS and TD cohorts as they performed face or visuospatial tasks in the scanner, AFNI’s 3dttest++ was again used across the whole brain to model group differences with a general linear regression model that controlled for chronological age and sex effects. Once statistical maps were calculated between groups for each of the four tasks, the voxelwise Z-scores from the two face processing and from the two visuospatial processing tasks, respectively, were combined using Stouffer’s Z method^73^. This method generates a meta-analytic Z-score for each voxel that represents between-group task activation differences combined from each of the two domain-specific tasks (face and visuospatial processing). Because the results of this approach could be influenced by voxels that were strongly significant in just one of the two tasks alone, we included only those voxels that showed significance at p_uncorrected_ < 0.05 in both tasks. The resulting data were thresholded at a voxelwise significance of *p* < 0.001 and corrected for multiple comparisons with family-wise-error (FWE) methods at *p* < 0.05 corrected, using 3dClustSim to compute a cluster threshold based on 100,000 Monte Carlo simulations of synthesized white Gaussian noise, taking into account the smoothing and resampling characteristics of the underlying data using the ACF method^74^.

### Ethics declarations

All participants with WS, healthy volunteers, and their parent(s)/legal guardians provided informed consent prior to all procedures and data collection. Children and adolescents also provided assent. These consent processes and all procedures were carried out in accordance with the NIH Institutional Review Board guidelines and with the Declaration of Helsinki. Data were collected under NIH protocol 10M0112/ NCT01132885.

## Results

### Activation during face processing and visuospatial tasks in typically developing children

To investigate whether expected regions were appropriately activated by the face processing and visuospatial tasks in TD children and adolescents, we first tested for within-group activation patterns in the TD group alone. For both the Tetris and the Spatial Location Tasks, which targeted visuospatial processing, we found robust engagement of the dorsal stream bilaterally, including maximal activation in the IPS bilaterally (Fig. 1, top row). For both the Match-to-Sample Face Task and the One-Back Face Matching Task, which targeted face processing, we observed strong engagement of the ventral stream bilaterally, including maximal activation in the fusiform gyri bilaterally (Fig. 1, bottom row).

### Altered activation during face processing and visuospatial tasks in Williams syndrome

We next identified within-group activation patterns for people with WS for each of the four tasks separately (Supplementary Fig. 1, Supplementary Tables 1–4). Similar regions were activated in both groups for each task. Specifically, both visuospatial processing tasks showed engagement of the bilateral dorsal stream, but with less robust activation for the WS group than for TDs; and both face processing tasks showed engagement of the ventral stream bilaterally, particularly of the bilateral fusiform gyri, but with more robust activation for the WS group.

**Figure 2:**
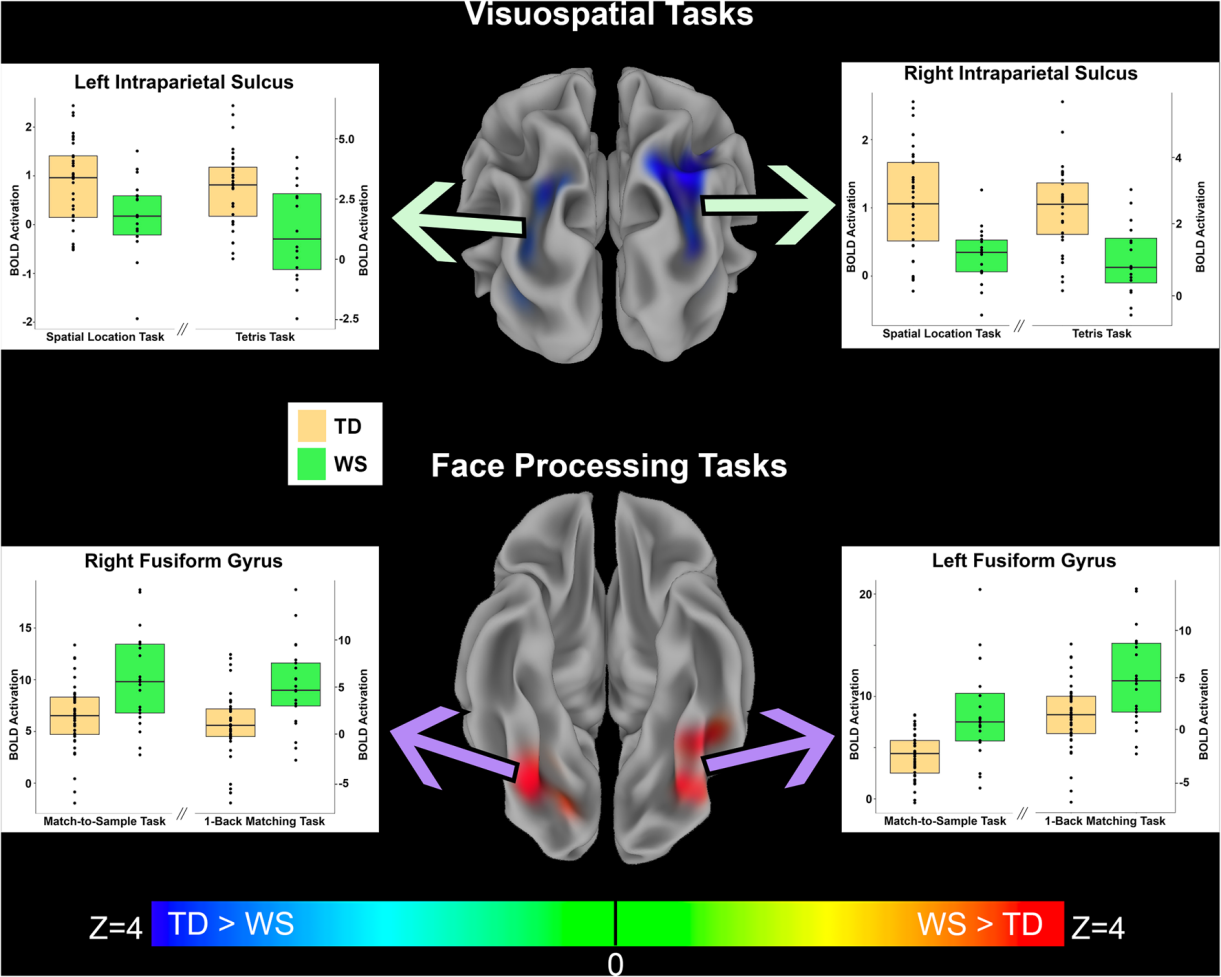
Functional activation differences between children with WS compared to typically developing children in response to visuospatial tasks and face processing tasks. *Top row* Middle image displays a posterior view of the inflated brain surface with overlaid between-group meta-Z statistics of the two visuospatial tasks combined, showing bilateral hypoactivation of the intraparietal sulcus (IPS) in participants with WS (TD > WS, *p* < 0.001 FWE corrected). Green arrows point to plots of the average extracted BOLD from the identified significant IPS clusters for individuals with WS and TD controls for both visuospatial processing tasks, separately. Plots show that TD participants had greater activation than children with WS in the Spatial Location Task and the Tetris Task in both the right and left IPS regions. *Bottom row* Middle image displays an inferior view of the inflated brain surface with the overlaid color representing the between-group meta-Z statistics for both face processing tasks combined, showing bilateral hyperactivation of the fusiform gyri in children with WS (WS > TD, *p* < 0.001 FWE corrected). Purple arrows point to plots of the average extracted BOLD from the identified significant fusiform clusters for individuals with WS and TD controls for the two face processing tasks separately. Plots show that children with WS had greater activation than TD children in both the Match-to-Sample Face Task and the One-Back Face Matching Task in both the right and left fusiform regions.

**Figure 3:**
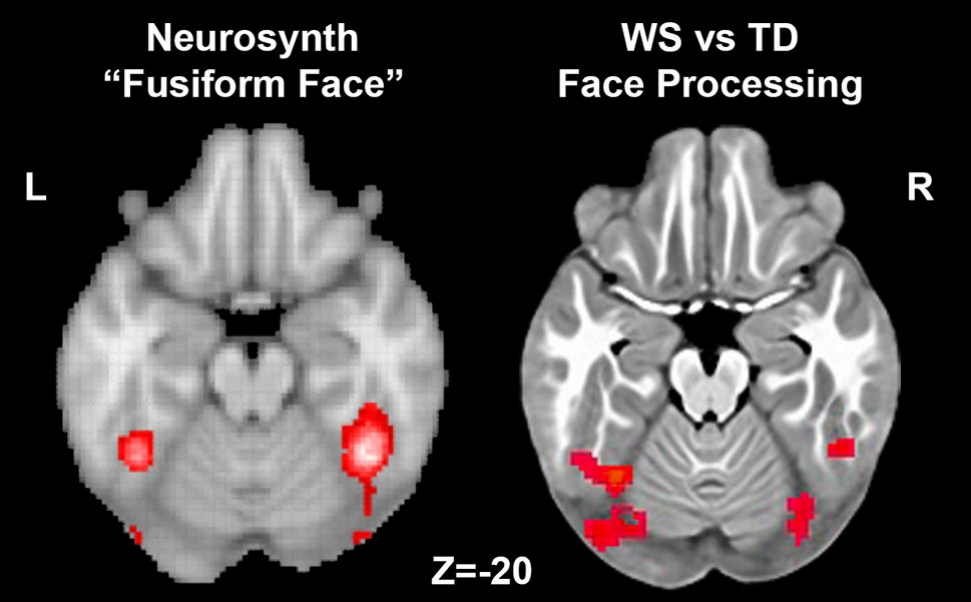
Comparison of Neurosynth meta-analysis of “fusiform face” term with regions showing increased BOLD activation during face processing in children with WS in the present study. *Left* an axial brain slice at z = − 20 MNI coordinate with red colors representing the regions showing significant association (Z-statistic > 10) with the term “fusiform face” in 143 published studies. *Right* an axial brain slice, also at z = − 20, with red colors representing regions in the present study showing hyperactivation in WS during the combined meta-Z analysis of the two face processing tasks studied. Note the high degree of overlap between the left and right fusiform areas of activation. Neurosynth image was accessed from http://www.neurosynth.org.

To test whether activation in response to these tasks was altered in children and adolescents with WS, we conducted a between-groups analysis for each task separately (Supplementary Fig. 1) and then combined the results across each pair of domain-specific paradigms to create “meta-Z-score” maps for each domain (i.e., visuospatial or face processing). In response to tasks targeting visuospatial function, we found that individuals with WS had decreased activation in the IPS bilaterally compared to typically developing peers (Fig. 2; Table 2). In response to tasks targeting face processing, individuals with WS showed increased activation in the fusiform gyri bilaterally compared to typically developing children (Fig. 2; Table 3). To better characterize this finding, we compared its location and extent with the representation of the fusiform face area from the online Neurosynth term-based meta-analytic database^75^ and noted a high degree of overlap (Fig. 3). Additionally, the combined analysis of the face processing tasks showed that individuals with WS also have increased activation of the bilateral premotor area, right superior parietal lobule, and right middle occipital gyrus (Table 3).

**Table 2:** Clusters showing significant between-group activation differences in the TD versus WS combined meta-analytic analysis of the two visuospatial processing tasks.

Cluster peak	X	Y	Z	Max Z-statistic	# Voxels
Right intraparietal sulcus	+ 25	− 71.8	+ 53	5.4	883
Left occipital region	− 32.5	− 84.2	+ 10.4	4.2	130
Left intraparietal sulcus	− 20	− 74.2	+ 50.5	4.3	112

Positive Z-statistics indicate TD > WS activation. No findings in the opposite direction (WS > TD activation) were observed. Coordinates are reported in MNI space.

**Table 3:** Clusters showing significant between-group activation differences in the TD versus WS combined meta-analytic analysis of the two face processing tasks.

Cluster peak	X	Y	Z	Max Z-statistic	# Voxels
Left fusiform gyrus	− 35	− 61.8	− 22	− 5.2	391
Right fusiform gyrus	+ 35	− 79.2	− 14.5	− 5.5	324
Left premotor area	− 25	− 4.2	+ 53.0	− 4.8	203
Right middle occipital gyrus	+ 40	− 81.8	+ 13.0	− 5.8	168
Right superior parietal lobule	+ 20	− 61.8	+ 60.5	− 4.6	121
Right premotor area	+ 25	+ 0.8	+ 53	− 5.0	104

Negative Z-statistics indicate WS > TD activation. No findings in the opposite direction (TD > WS activation) were observed. Coordinates are reported in MNI space.

Next, from the most significant clusters identified from the “meta-Z” between-groups analyses that combined the two task paradigms for each cognitive domain (i.e., fusiform cluster for the face processing tasks and parietal cluster for the visuospatial processing tasks), we extracted average BOLD activation values for each separate task to ensure the findings were truly driven by greater/lesser activation, rather than relative deactivation or the inclusion of regions not used by TD participants in the task activation maps (Fig. 2, box plots). For the bilateral IPS clusters identified from the between-group “meta-Z” analysis of combined visuospatial tasks, individuals with WS exhibited lower activation than TD participants in response to both visuospatial tasks analyzed separately (Fig. 2, top box plots). For the bilateral fusiform clusters, the results for each task analyzed separately also agreed with the results of the between-group “meta-Z” analysis of the two combined face processing tasks: activation for the cohort with WS was more robust than for the TD cohort (Fig. 2, bottom box plots).

**Figure 4:**
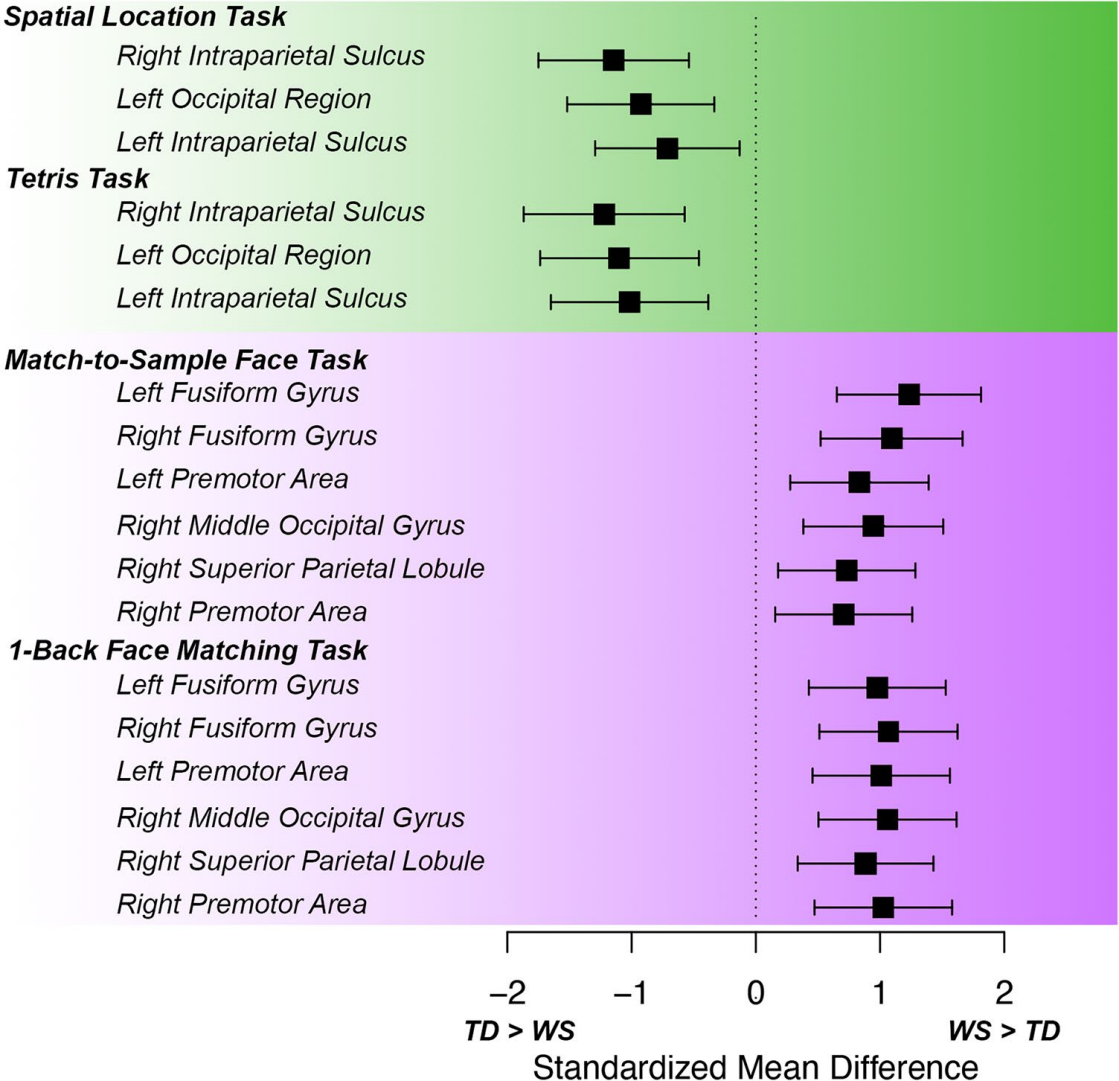
Standardized BOLD activation differences between WS and TD groups derived from each significant cluster in the between-groups combined meta-Z analyses. Clusters were derived from combined analyses of visuospatial or face processing tasks (see Tables 2 and 3) and extracted from each task individually. Green indicates tasks that targeted visuospatial processing, and purple indicates tasks that targeted face processing. X-axis shows standardized mean activation difference between WS and TD groups, with negative numbers representing greater activation in TD children and positive numbers representing greater activation in children with WS. Lines for each cluster represent the 95% confidence interval of the standardized mean difference between groups. Note that the difference in BOLD activation consistently indicates greater activation in TD children than children with WS during visuospatial tasks and in children with WS than in TD children during face processing tasks.

In addition to the results for the main regions of interest, the fusiform and IPS, we also examined the differences in BOLD activation for every cluster identified in the between-group “meta-Z” analyses that combined tasks within cognitive domains. During visuospatial tasks, TD children showed greater activation than children with WS in all regions, while children with WS consistently showed greater activation during face processing tasks (Fig. 4).

It is interesting to note that while our face processing tasks evoked significant neural activation in face processing areas within the bilateral fusiform gyri as expected, there was also robust activation of other known face processing areas, such as the amygdala, within both TDs and individuals with WS during both face processing tasks (Supplementary Tables 1 and 2). However, in contrast to the prominent between-group differences observed in the fusiform gyri (greater activation in WS), we did not find between-group differences in amygdala activation during face processing, even when explored at an uncorrected threshold of *p* = 0.01.

### Effects of potential confounds on brain activation patterns

#### Task performance

To explore whether the activation differences were driven by task performance differences between WS and TD groups, we determined accuracy for each individual for each task. For all tasks, children and adolescents with WS were less accurate than the TD cohort: for the Match-to-Sample Face Task, 91.5% vs. 97.9% *p* = 0.001; for the One-Back Face Matching Task, 84.4% vs. 92.4%, *p* = 0.004; for the Spatial Location Task, 69.2% vs. 78.5%, *p* = 0.0002; and for the Tetris Task, 62.1% vs. 86.7%, *p* = 1 × 10^–8^ (Supplementary Fig. 2). To test whether the between-group activation differences were driven by task performance, we repeated our fMRI analyses with a subset of participants from each group (i.e., WS and TD) who were individually matched for task performance (Table 4). In this between-group analysis, we found that the voxelwise T-statistics for the performance-matched subgroup comparisons for each task were highly correlated with the voxelwise statistics for the entire sample (Tetris Task R = 0.82, Spatial Location Task R = 0.77, Match-to-Sample Face Task R = 0.81, One-Back Face Matching Task R = 0.90; all *p* values < 0.000001), with between-group differences that recapitulated the fusiform and IPS findings of the larger sample.

**Table 4:** Task accuracy of performance-matched groups for each of the four fMRI task paradigms.

Task category	Task name	Participant group	n	Accuracy (%)	*p* value (WS versus TD)
Face Processing	Match-to-Sample Face Task	Williams syndrome	16	96	0.85
		Typically developing	16	97	
	One-back Face Matching Task	Williams syndrome	18	88	0.77
		Typically developing	18	89	
Visuospatial Construction	Tetris Task	Williams syndrome	11	67	0.13
		Typically developing	11	74	
	Spatial Location Task	Williams syndrome	15	72	0.99
		Typically developing	15	72	

Each participant with Williams syndrome was matched as closely as possible to a typically developing participant based on task accuracy.

#### General cognitive abilities

Next, to determine whether our results were driven by group differences in general cognitive abilities, we tested whether the between-group activation differences were affected by differences in general cognition by repeating the between-group analyses with age, sex, and K-BIT composite IQ score as covariates. The voxelwise T-statistics across all gray matter voxels were highly correlated between results with and without IQ as a covariate (Tetris Task R = 0.99, Spatial Location Task R = 0.97, Match-to-Sample Face Task R = 0.99, One-Back Face Matching Task R = 0.98; all *p* values < 0.000001). In particular, our central between-group fMRI findings in the bilateral fusiform and IPS held when controlling for IQ, suggesting that the between-group fMRI differences in activation patterns were not driven by differences in general cognitive ability.

#### Combined effects of age, sex, and IQ on performance

To determine whether age, sex, or IQ impacted task performance, we employed regression models. Neither IQ nor sex was significantly related to performance for any task (uncorrected *p*’s > 0.05 in all tasks for both WS and TD groups), though age and performance were significantly related in two tasks for the TD group (Tetris Task and One-Back Face Matching Task) and one task in the WS group (One-Back Face Matching Task) when correcting for analysis of three variables. See Supplementary Table 5.

## Discussion

Here, we show that children and adolescents with WS have altered neural responses in the dorsal visual processing stream when undertaking both visuospatial tasks and in the ventral visual processing stream during face processing tasks. Specifically, we demonstrated reduced engagement of the bilateral IPS in children and adolescents with WS during visuospatial processing but increased activation in the bilateral fusiform gyri in response to face stimuli. These findings recapitulate at the neural level two core clinical observations in both children and adults with WS—visuospatial deficits and heightened attention to faces.

Diminished IPS BOLD response during the tasks involving visuospatial stimuli in our sample of young people with WS precisely aligns with our prior observations of reduced IPS neural engagement during spatial location matching in an adult WS cohort^39^. Unequivocally heightened responsivity of specialized fusiform cortex regions to face processing tasks in the present group of children and adolescents with WS (Fig. 3) lends clarity to a neuroimaging literature that has not consistently observed face-related neurofunctional differences despite clearly suggestive behavioral and clinical observations of heightened attention to social stimuli. Importantly, for each stimulus type in our experiments, findings were replicable across two independent tasks that share a common cognitive operation of interest (visuospatial or face processing), but also have divergent additional cognitive demands: comparison of two stimuli presented in series with a memory component (One-Back Face Matching Task, Spatial Location Task) or multifaceted matching judgments based on evaluation of more than one option without recall demands (Match-to-Sample Face Task, Tetris Task). Because IPS and fusiform observations were consistent despite these substantive differences in how visuospatial and face information was used across paradigms, they may represent generalizable visual object processing alterations during spatial/facial processing in WS.

The contrasting findings regarding engagement of visuospatial versus face processing networks is consistent with recent resting-state fMRI findings in children and adolescents with WS, in which the IPS was found to have increased functional connectivity with brain regions subserving social processing and decreased connectivity with regions subserving visuospatial processing^47^. Archeological studies have suggested that this potential visuospatial-social processing balance seen in WS may have evolutionary origins. For example, endocranial analyses of Neanderthal skulls show that, compared to modern humans, neural resources devoted to visuospatial processing in Neanderthal brains may have been increased at the expense of social processing^1^. The current findings further support the notion that, as the brain has a finite supply of resources to distribute^76^, an evolutionary balance may exist between visuospatial and social functioning, whereby increasing the resources devoted toward one behavior leads to a reciprocal decrease in the other, with the behavioral and brain phenotypes observed in WS fitting well in this speculative framework. Of further evolutionary interest, although the IPS is present in all modern primate species, it has undergone substantial expansion in humans, perhaps related to their enhanced visuospatial abilities^77^. It also is notable that the fusiform gyrus is only found in hominids^78^.

The results presented here also shed further light on the neural development of ventral and dorsal stream regions underlying visuospatial and face processing. Previous functional imaging studies of these two key domains of the WS profile have predominantly focused on adults with WS, so it was unclear whether the WS-associated differences seen on brain imaging are present before adulthood or if they emerge over time, perhaps due to a lifetime of living with the 7q11.23 CNV. This knowledge gap is underscored by the fact that both parietal and fusiform activation during visuospatial and face processing tasks, respectively, show substantial maturational changes over childhood and adolescence in the general population^79,80^. The current findings confirm that functional changes in both the IPS and the fusiform gyrus are already present in children and adolescents with WS, and their foundations likely occur even earlier. Importantly, because prior behavioral work has raised the interesting possibility of differential developmental trajectories of dorsal and ventral stream abilities in TD children^2,3^ (though conflicting data exist^81^), it may be particularly informative to quantify both of these neurofunctional signatures in parallel longitudinally over development in WS.

Because our observations were made in children and adolescents with WS, it is possible that over-engagement of face processing circuits from a young age could influence development of these circuits, which might occur via multiple potential mechanisms that depend on network activity, including myelination^82^ and known synaptic plasticity mechanisms^83^. While future work will be needed to better delineate both the specific subregional contours and developmental trajectories of ventral and dorsal stream differences in WS, the present work solidifies the importance of one or more 7q11.23 WS critical region genes in visuospatial and face processing and provides a platform for further work to elucidate specific neurogenetic contributors to the types of dorsal and ventral stream alterations seen in WS^84^. Additionally, this work is unable to identify which of the WS critical region genes is/are involved in the behavioral phenotypes in WS. Prior experimentation has implicated the *LIMK1* gene in IPS structural and functional findings^47,84^. Others have reported that the *GTF2I* gene is important in shaping the WS hypersocial phenotype^85^, and may act in part through effects on neuronal myelination^86,87^. Future studies will be needed to determine whether these genes may differentially drive neurodevelopment and functional activation outcomes as measured here.

It is worth noting that the necessarily small sample sizes in this rare neurogenetic condition is a challenge that future work should aim to alleviate through replication in independent cohorts. Additionally, future work is needed to test the present neuroimaging results by employing a wider array of tasks so that common or specific cognitive features underlying our findings can be more specifically delineated. Further, the age range of participants and the cross-sectional nature of the data presented here preclude a full picture of genetically-mediated developmental differences in brain networks subserving visuospatial and social/facial processing, a compelling question that will require younger samples and longitudinal neuroimaging studies. Here, to help mitigate the possibility of age effects on our results, an especially important consideration for studies of children and adolescents, we have matched our TD and WS samples by chronological age and have controlled for this variable in our analyses. Nonetheless, future work investigating developmental trajectories will be important to further understand neuroimaging findings in WS during this critical period.

In summary, we present new evidence that even in childhood and adolescence, individuals with WS already show both reduced dorsal stream engagement during visuospatial processing and concomitant ventral stream hyperactivation in response to tasks requiring face processing. These genetically-associated, contrasting neural phenotypes are robust to divergent task-dependent cognitive demands and, thus, provide important markers to guide the search for specific gene-brain relationships, not only in WS, but also to better understand sources of variability in the general population. Finally, these data provide a clear example that neurogenetic mechanisms can bias the function of neural circuits, thereby affecting behavioral traits.

### Supplementary Information

Supplementary Information.

## Data Availability

The datasets for individual participants generated and/or analyzed during the current study are not publicly available due to NIH Institutional Review Board (IRB) restrictions because of privacy concerns for this rare cohort. Summary data may be available from the corresponding author on reasonable request that is consistent with IRB restrictions.
