# Supplementary material for: Contrasting neurofunctional correlates of face- and visuospatial-processing in children and adolescents with Williams syndrome: convergent results from four fMRI paradigms
Source: Sci Rep. 2024 May 5;14:10304. doi: 10.1038/s41598-024-60460-5 (PMC11070425; doi:10.1038/s41598-024-60460-5)
Supplement: Supplementary file 1 — Supplementary Information. [file 41598_2024_60460_MOESM1_ESM.pdf]

Contrasting Neurofunctional Correlates of Face- and Visuospatial-Processing in Children and Adolescents with Williams Syndrome: Convergent Results from Four fMRI Paradigms

Madeline H. Garvey, Tiffany Nash, J. Shane Kippenhan, Philip Kohn, Carolyn B. Mervis, Daniel P. Eisenberg, Jean Ye, Michael D. Gregory, Karen Faith Berman

Included Supplementary Information:

**Supplementary Figure 1**	**Functional activation by task in typically developing individuals and people with WS**	**Page 2**
**Supplementary Figure 2**	**Performance accuracy for all participants during each of the four fMRI task**	**Page 3**
**Supplementary Table 1**	**Clusters showing significant activation in each group and between-group differences for the Match-to-Sample Face Task**	**Page 4**
**Supplementary Table 2**	**Clusters showing significant activation in each group and between-group differences in the One-Back Face Matching Task**	**Page 5**
**Supplementary Table 3**	**Clusters showing significant activation in each group and between-group differences in the Spatial Location Task**	**Page 6**
**Supplementary Table 4**	**Clusters showing significant activation in each group and between-group differences in the Tetris Task**	**Page 7**
**Supplementary Table 5**	**Partial correlation coefficients between age, IQ, sex and performance separated by task**	**Page 8**

Supplementary Figure 1. Functional activation by task in typically developing individuals and people with WS. Top two rows: activation patterns shown separately for each of two visuospatial tasks within TD individuals (left column) and within people with WS (middle column), as well as between-group activation difference maps for each task (right column). For each visuospatial processing task, TD individuals and people with WS activated the dorsal stream and, in particular, the bilateral intraparietal sulci, with more robust engagement seen in TD individuals (blue voxels in the between-groups analysis). **Bottom two rows:** activation patterns shown separately for each of two face processing tasks within TD individuals (left column) and within people with WS (middle column), as well as between-group activation difference maps for each task (right column). For each face processing task, both groups (TD individuals and people with WS) activated the ventral stream and, in particular, the bilateral fusiform gyri, with more robust engagement in particular fusiform areas in children and adolescents with WS (red voxels in the between-groups analysis). Activation maps are shown thresholded at $p < 0.05$, family-wise error corrected.

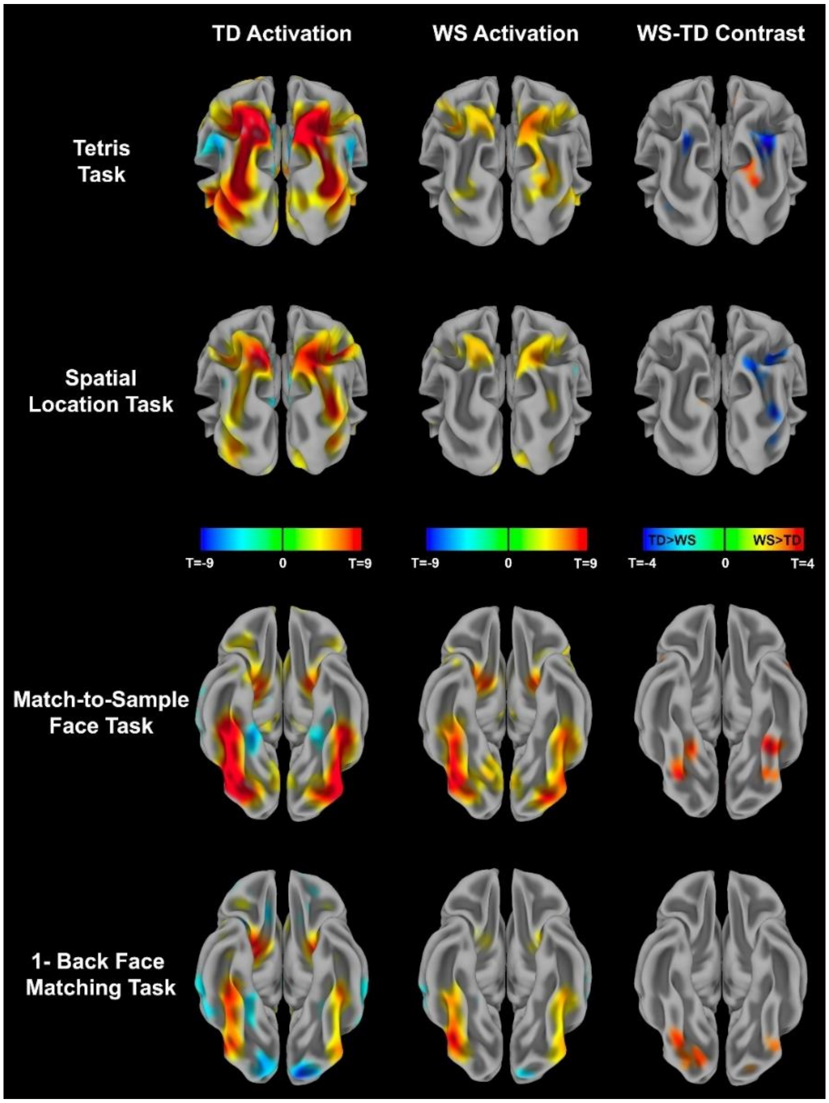

Supplementary Figure 2: Performance accuracy for all participants during each of the four fMRI tasks. Dots represent accuracy for each individual, boxes represent the range of the central 50% of data, and whiskers represent the spread of the maximum and minimum values for each group within 1.5x the interquartile range. TD=typically developing individuals, WS=individuals with Williams syndrome.

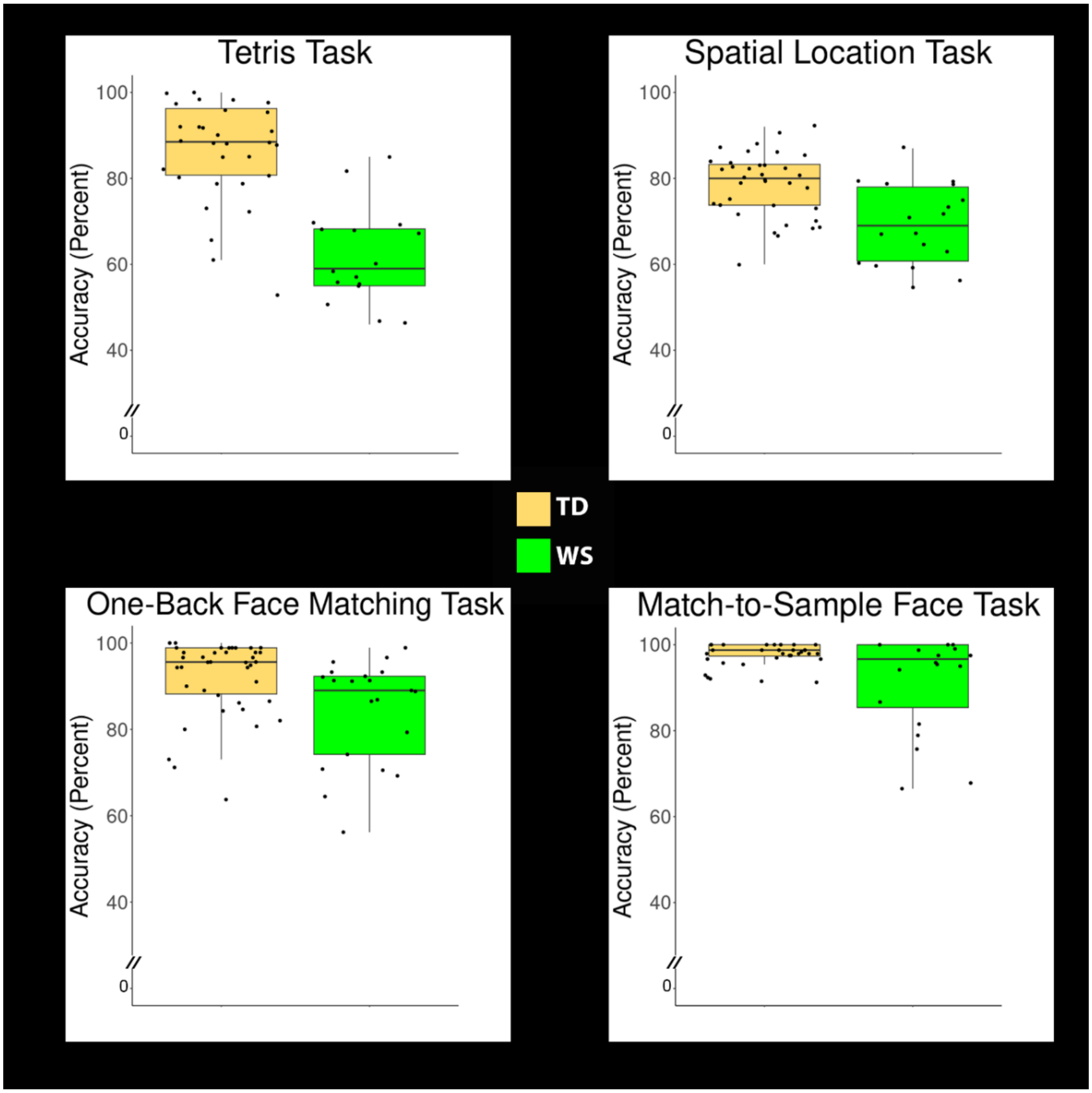

Supplementary Table 1. Clusters showing significant activation in each group and between-group differences for the Match-to-Sample Face Task (BA = Brodmann Area)

TD participants

Location of Peak T Statistic in Cluster	Peak BA	X_{MNI}	Y_{MNI}	Z_{MNI}	Max T	# Voxels
Right Fusiform Gyrus	37	42.5	-51.8	-24.5	22.6	6750
Right Amygdala	N/A	20.0	-6.8	-14.5	9.9	2170
Right Premotor Cortex	6	47.5	3.2	55.5	6.8	869
Left Precentral Gyrus	4	-47.5	-24.2	65.5	6.3	208
Right Orbitofrontal Cortex	11	2.5	53.2	-24.5	6.1	246
Left Inferior Frontal Gyrus	47	-40.0	25.8	-19.5	5.6	107
Right Superior Temporal Gyrus	22	45.0	-41.8	15.5	5.2	112
Right Fusiform Gyrus	37	25.0	-51.8	-12	-6.9	209
Left Fusiform Gyrus	37	-27.5	-54.2	-12.0	-6.4	140
Right Superior Temporal Gyrus	22	67.5	-19.2	0.5	-5.1	346

WS participants

Location of Peak T Statistic in Cluster	Peak BA	X_{MNI}	Y_{MNI}	Z_{MNI}	Max T	# Voxels
Right Fusiform Gyrus	37	40.0	-81.8	-12.0	15.4	6031
Right Amygdala	N/A	17.5	-6.8	-14.5	9.2	576
Left Amygdala	N/A	-30.0	-1.8	-24.5	9.2	412
Left Frontal Eye Fields	8	-42.5	15.8	25.5	7.5	178
Left Thalamus	N/A	-20.0	-29.2	-2.0	7.1	230
Right Temporal Pole	38	47.5	15.8	-34.5	6.8	204
Right Frontal Eye Fields	44	45.0	18.2	25.5	6.4	163
Left Orbitofrontal Cortex	11	-5.0	53.2	-22.0	6.3	138
Left Pars Orbitalis	47	-45.0	28.2	-9.5	5.6	154
Left Motor Cortex	4	-37.5	-16.8	70.5	5.5	188
Left Medial Temporal Gyrus	21	-50.0	-41.8	5.5	5.2	153

Between-Group TD – WS

Location of Peak T Statistic in Cluster	Peak BA	X_{MNI}	Y_{MNI}	Z_{MNI}	Max T	# Voxels
Left Visual Association Area	18	-15.0	-96.8	3.0	-5.7	168
Right Posterior Cingulate Cortex	18	25.0	-64.2	18.0	-5.2	406
Left Fusiform Gyrus	37	-32.5	-56.8	-19.5	-4.8	194
Right Fusiform Gyrus	44	35.0	-76.8	-12.0	-4.4	251
Left Superior Temporal Gyrus	37	-55.0	-39.2	8.0	-4.3	326
Left Temporal Pole	38	-52.5	0.8	-27.0	-4.2	156
Right Middle Occipital Gyrus	19	40.0	-79.2	13.0	-4.0	123
Right Superior Parietal Lobule	7	27.5	-49.2	63.0	-3.9	186

Supplementary Table 2. Clusters showing significant activation in each group and between-group differences in the One-Back Face Matching Task (BA=Brodmann Area)

TD Participants

Location of Peak T Statistic in Cluster	Peak BA	X_{MNI}	Y_{MNI}	Z_{MNI}	Max T	# Voxels
Right Amygdala	NA	25.0	-1.8	-22.0	10.2	3218
Left Hippocampus	NA	-27.5	-6.8	-22.0	8.8	1258
Left Medial Prefrontal Cortex	10	2.5	60.8	-14.5	7.5	362
Left Angular Gyrus	39	-60.0	-69.2	15.5	5.8	382
Right Inferior Frontal Gyrus	45	57.5	33.2	5.5	5.4	136
Right Posterior Cingulate Cortex	23	5.0	-56.8	25.5	5.2	302
Right Primary Auditory Cortex	41	42.5	-16.8	8.0	4.4	115
Left Superior Parietal Lobule	7	-25.0	-64.2	55.5	-10.6	2545
Left Brodmann Area 18	18	-10.0	-101.8	-17.0	-10.0	211
Right Superior Parietal Lobule	7	27.5	-69.2	53.0	-9.9	2632
Right Premotor Cortex	6	27.5	3.2	50.5	-9.2	2201
Right Insula	13	32.5	20.8	3.0	-8.4	372
Left Orbitofrontal Cortex	11	-22.5	53.2	-17.0	-7.9	212
Left Insula	13	-32.5	23.2	-4.5	-7.5	301
Right Visual Association Area	18	20.0	-101.8	-12.0	-6.6	351
Right Fusiform Gyrus	37	57.5	-54.2	-14.5	-6.5	446
Right Fusiform Gyrus	37	27.5	-56.8	-12.0	-5.9	161
Right Premotor Cortex	6	47.5	3.2	25.5	-5.9	145
Left Cingulate Gyrus	23	-7.5	-29.2	25.5	-5.7	185
Right Prefrontal Cortex	10	42.5	53.2	5.5	-5.6	563
Left Prefrontal Cortex	10	-37.5	58.2	13.0	-5.6	240
Left Fusiform Gyrus	37	-62.5	-44.2	-22.0	-5.5	234
Left DLPFC	9	-42.5	25.8	35.5	-5.1	130
Right Orbitofrontal Cortex	11	17.5	68.2	-19.5	-5.0	182

WS Participants

Location of Peak T Statistic in Cluster	Peak BA	X_{MNI}	Y_{MNI}	Z_{MNI}	Max T	# Voxels
Right Fusiform Gyrus	37	40.0	-81.8	-14.5	10.2	831
Left Fusiform Gyrus	37	-42.5	-91.8	-4.5	8.3	843
Left Amygdala	NA	-27.5	-1.8	-19.5	6.0	128
Left Angular Gyrus	39	-45.0	-54.2	48.0	-6.6	773
Right Superior Parietal Lobule	7	20.0	-81.8	53.0	-6.3	121

Between-Group TD – WS

Location of Peak T Statistic in Cluster	Peak BA	X_{MNI}	Y_{MNI}	Z_{MNI}	Max T	# Voxels
Right Middle Occipital Gyrus	19	40.0	-86.8	10.5	-5.6	1478
Right Premotor Area	6	27.5	0.8	48.0	-5.2	311
Left Superior Parietal Lobule	7	-25.0	-64.2	55.5	-5.1	841
Left Premotor Area	6	-25.0	-6.8	50.5	-5.0	322
Left Fusiform Gyrus	37	-40.0	-84.2	-17.0	-3.6	102
Right Visual Association Area	19	57.5	-64.2	20.5	4.1	107

Supplementary Table 3. Clusters showing significant activation in each group and between-group differences in the Spatial Location Task (BA = Brodmann Area)

TD Participants

Location of Peak T Statistic in Cluster	Peak BA	X_{MNI}	Y_{MNI}	Z_{MNI}	Max T	# Voxels
Left Superior Parietal Lobe	7	-15.0	-66.8	55.5	11.4	8860
Left Premotor Cortex	6	-30.0	-6.8	55.5	10.6	445
Right Premotor Area	6	27.5	5.8	50.5	8.2	1583
Right Premotor Area	8	5.0	28.2	43.0	7.3	202
Right Inferior Frontal Gyrus	47	42.5	53.2	-17.0	6.7	216
Left Premotor Area	6	-2.5	20.8	48.0	6.4	104
Right Anterior Insula	13	30.0	23.2	-2.0	6.1	225
Left Premotor Area	6	-57.5	5.8	38.0	5.1	105
Right Fusiform Gyrus	37	57.5	-56.8	-9.5	4.8	306
Left Angular Gyrus	39	-47.5	-76.8	40.5	-7.2	118
Right Anterior Cingulate Cortex	32	7.5	38.2	-2.0	-6.4	949
Left Frontal Eye Fields	8	-22.5	30.8	43.0	-6.4	356
Posterior Cingulate Cortex	23	-2.5	-56.8	15.5	-5.7	469
Left Medial Temporal Gyrus	21	-57.5	-11.8	-22.0	-5.0	103

WS Participants

Location of Peak T Statistic in Cluster	Peak BA	X_{MNI}	Y_{MNI}	Z_{MNI}	Max T	# Voxels
Right Superior Parietal Lobe	7	20.0	-66.8	55.5	7.7	776
Right Premotor Area	6	22.5	-1.8	55.5	7.7	309
Right Middle Occipital Gyrus	18	25.0	-91.8	5.5	6.6	940
Left Superior Parietal Lobe	7	-20.0	-61.8	50.5	6.6	598
Left Fusiform Gyrus	37	-22.5	-94.2	-17.0	5.7	194
Right Medial Prefrontal Cortex	10	2.5	55.8	0.5	-5.4	138

Between-Group TD – WS

Location of Peak T Statistic in Cluster	Peak BA	X_{MNI}	Y_{MNI}	Z_{MNI}	Max T	# Voxels
Right Middle Occipital Gyrus	19	37.5	-81.8	20.5	4.3	186
Right Intraparietal Sulcus	7	20.0	-71.8	55.5	4.1	184
Right Superior Parietal Lobule	7	37.5	-41.8	43.0	3.8	163

Supplementary Table 4. Clusters showing significant activation in each group and between-group differences in the Tetris Task (BA = Brodmann Area)

TD Participants

Location of Peak T Statistic in Cluster	Peak BA	X_{MNI}	Y_{MNI}	Z_{MNI}	Max T	# Voxels
Left Superior Parietal Lobe	7	-20.0	-69.2	55.5	17.8	17222
Right Premotor Cortex	6	25.0	0.8	60.5	13.9	1994
Right Thalamus	NA	15.0	-26.8	13.0	8.5	1505
Left Insula	13	-32.5	23.2	-4.5	8.0	296
Right DLPFC	9	42.5	35.8	20.5	7.7	536
Right Premotor Area	8	5.0	18.2	45.5	7.7	438
Right Orbitofrontal Cortex	11	20.0	53.2	-19.5	6.7	164
Right Anterior Cingulate Cortex	24	5.0	5.8	28.0	6.3	101
Left Premotor Cortex	6	-2.5	13.2	48.0	6.1	120
Left DLPFC	9	-50.0	30.8	33.0	5.3	122
Left Angular Gyrus	39	-52.5	-71.8	40.5	-9.6	697
Right Angular Gyrus	39	57.5	-64.2	35.5	-9.3	395
Right Posterior Cingulate Cortex	23	7.5	-51.8	28.0	-7.4	808
Left Anterior Cingulate Cortex	32	-2.5	33.2	3.0	-6.6	1718
Right Superior Temporal Gyrus	41	62.5	-11.8	-12.0	-5.5	151
Right Frontal Eye Fields	8	20.0	38.2	43.0	-5.4	197
Left Medial Temporal Gyrus	21	-50.0	-9.2	-19.5	-5.4	120

WS Participants

Location of Peak T Statistic in Cluster	Peak BA	X_{MNI}	Y_{MNI}	Z_{MNI}	Max T	# Voxels
Right Premotor Area	6	27.5	-6.8	50.5	9.7	524
Right Visual Association Area	19	40.0	-86.8	10.5	9.3	3820
Right Thalamus	NA	5.0	-14.2	-2.0	7.4	230
Left Premotor Area	6	-47.5	0.8	33.0	6.5	179
Right Premotor Area	6	50.0	8.2	28.0	6.4	142

Between-Group TD – WS

Location of Peak T Statistic in Cluster	Peak BA	X_{MNI}	Y_{MNI}	Z_{MNI}	Max T	# Voxels
Right Intraparietal Sulcus	7	27.5	-71.8	48.0	4.8	377
Left Visual Association Area	18	-22.5	-79.2	3.0	4.8	336
Right Retrosplenial Cortex	30	32.5	-51.8	5.5	4.8	161
Left Intraparietal Sulcus	7	-22.5	-71.8	48.0	4.3	119
Left Prefrontal Cortex	10	-25.0	68.2	8.0	-4.6	101
Right Parieto-Occipital Junction	19	15.0	-84.2	38.0	-4.4	116

Supplementary Table 5. Partial correlation coefficients between age, IQ, sex and performance separated by task. * represents partial correlation coefficients with a corresponding p value of < 0.05, uncorrected, ** represents partial correlation coefficients with a corresponding p value of < 0.05, corrected for multiple comparisons (for three measures per task).

Effects of Age, IQ, and Sex on Performance in Typically Developing Cohort

	Spatial Location Task	Tetris Task	Match-to-Sample Face Task	1-Back Face Matching Task
**Age**	0.42*	0.57**	0.17	0.49**
**IQ**	0.35	0.3	0.2	0.29
**Sex**	0.22	0.1	0.08	-0.15

Effects of Age, IQ, and Sex on Performance in Williams Syndrome Cohort

	Spatial Location Task	Tetris Task	Match-to-Sample Face Task	1-Back Face Matching Task
**Age**	0.2	0.14	0.55*	0.63**
**IQ**	-0.15	0.12	0.35	-0.14
**Sex**	0.36	0.29	-0.25	-0.35
